# PDK1/mTOR Signaling in Myeloid Cells Differentially Regulates the Early and Late Stages of Sepsis

**DOI:** 10.1155/2020/5437175

**Published:** 2020-07-25

**Authors:** Juan Du, Guoli Li, Mingxi Hua, Junyan Han, Yu Hao, Hui Zeng, Ang Li, Yaxian Kong

**Affiliations:** Beijing Key Laboratory of Emerging Infectious Diseases, Institute of Infectious Diseases, Beijing Ditan Hospital, Capital Medical University, Beijing 100015, China

## Abstract

The cecal ligation and perforation (CLP) model is the gold standard for the polymicrobial sepsis. In the CLP mice, the myeloid cells play an important role in septic shock. The phenotypes and the activation state of the macrophage and neutrophil correlate with their metabolism. In the present study, we generated the specific myeloid deletion of PDK1 and mTOR mice, which was the important regulator of metabolic signaling. We found that the deletion of PDK1 in the myeloid cells could aggravate the early septic shock in the CLP mice, as well as the deletion of mTORC1 and mTORC2. Moreover, PDK1 deletion attenuated the inflammation induced by LPS in the late stage on CLP mice, which was exacerbated in mTORC1 and mTORC2 knockout mice. Both PDK1 and mTORC1/2 could not only regulate the cellular metabolism but also play important roles on the myeloid cells in the secondary stimulation of sepsis. The present study will provide a theoretical prospect for the therapy of the septic shock in different stages.

## 1. Introduction

The cecal ligation and perforation (CLP) model is considered a gold standard of the experimental sepsis [1], which can imitate the clinical sepsis more accurately than the injection of endotoxin or purified bacteria. Sepsis is initiated by a systemic hyperinflammatory reaction that is named the early stage. Then, the inflammation shifts within a few days to a protracted anti-inflammatory and immunosuppressive state, named the late stage. During the late stage, the patients with the impaired immunity are more susceptible to secondary infections. The early sepsis in CLP has been confirmed with the transient systemic bacteremia and elevated cytokine levels in about the first five days, and the late stage has been confirmed with the enhanced peritoneal bacterial overgrowth and reduced circulating proinflammatory cytokines in the next five days [2]. There are different immune responses between the early and late stages of sepsis. Different regulatory factors may play important roles in the two stages. Therefore, it is particularly important to find out the key regulatory factors that may affect different stages of sepsis.

Myeloid cells were reported to aggravate the inflammation during the early stage of septic shock and also induce the immunosuppression with the risk to opportunistic infections in the late sepsis [3]. Myeloid cells could regulate the function of T cells and other innate immune cells in the early and late stages of sepsis. They can also secrete multiple inflammatory cytokines to induce septic shock in the early stage, such as TNF*α*, IL-6, IL-1*β*, and IFN-*γ*. Otherwise, in the late stage, myeloid cells inhibit the response of T cells and innate immune cells to the secondary infection through the immunosuppressive cytokines of high levels, such as IL-10 and TGF-*β*.

In the CLP mice, the myeloid cells, such as macrophages and neutrophils, play important roles in septic shock. The phenotype and activation of the macrophages and neutrophils correlate with their metabolic state [4]. So, the therapeutic modulation on the metabolism of myeloid cells may potentially improve the outcome of sepsis by altering the inflammatory responses leading to septic shock or immunotolerance. However, the effective target remains undefined. Phosphoinositide-dependent kinase-1 (PDK1) is the key regulator of phosphoinositide 3-kinase (PI3K) pathway. PDK1 can trigger phosphorylation of the key effectors downstream, such as the mammalian target of rapamycin (mTOR) [5]. The mTOR family involves two complexes: the mTORC1 and mTORC2. The mTORC1 is composed of mTOR, Raptor, Pras40, Deptor, and GBL/mLST8, while the mTORC2 is composed of mTOR, Rictor, mSin1, Proctor/PRR5, Deptor, and GBL/mLST8 [6]. PDK1/mTOR signaling is an important regulator within the metabolic signaling; thus, we wonder if PDK1/mTOR could be one target during sepsis. Therefore, we generated the mice of PDK1 and mTOR deletion specially on the myeloid system and analyzed the early shock and the late immunotolerance during sepsis.

## 2. Method

### 2.1. Mice

The mice with the myeloid deletion of PDK1 was achieved by crossing PDK1^fl/fl^ to LysM-Cre purchased from the Jackson Laboratory. Raptor^fl/fl^ and Rictor^fl/fl^ mice were purchased from the Jackson Laboratory. This line was backcrossed with C57BL/B6 mice for at least 8 generations before use in subsequent experiments, and all mice in the present study were the background of C57BL/6. All mice were at the age of 6–12 weeks and were randomly divided into different groups. The mice were bred and maintained in specific pathogen-free animal facilities, and all procedures involving animals were approved by the Animal Ethics Committee.

### 2.2. Primer

Primers for genotyping the mice were as follows:

*PDK1*: 5′-ATCCCAAGTTACTGAGTTGTGTTGGAAG-3′; 5′-TGTGGACAAACAGCAATGAACATACACGC-3′; and 5′-CTATGCTGTGTTACTTCTTGGAGCACAG-3′

*Raptor*: 5′-CTCAGTAGTGGTATGTGCTCA G-3′ and 5′-GGGTACAGTATGTCAGCACAG-3′

*Rictor*: 5′-CAAGCATCATGCAGCTCTTC-3′ and 5′-TCCCAGAATTTCCAGGCTTA-3′

*LysM-Cre*: 5′-CTTGGGCTGCCAGAATTTCTC-3′ and 5′-CCCAGAAATGCCAGATTACG-3′

Primers for mRNA level analysis were as follows:

*PDK1*: 5′-CTACCAGCCATGTCAGAGGATG-3′ and 5′-AGGCTGGTTTCCACCGTAGACA-3′

*Raptor*: 5′-CTTCCTATCCGTCTTGGCAGAC-3′ and 5′-CTCCAGACAGATGGCAATCAGG-3′

*Rictor*: 5′-CAGTGTGAGGTCCTTTCCATCC-3′ and 5′-GCCATAGATGCTTGCGACTGTG-3′

### 2.3. CD71 and CD98 Expression

For the analysis of surface CD71, CD98, and CD11b cells from the bone marrow by flow cytometry, the cells were stained with PBS containing 2% (wt/vol) FBS with fluorescein-labeled antibodies. Flow cytometry data were acquired on LSR Fortessa (BD) and analyzed with FlowJo. Their expression was presented as net mean fluorescence intensity (MFI). CD71 (R17217), CD98 (RL388), and KLRG1 (2F1, BD) antibodies were purchased from eBioscience and BD.

### 2.4. Western

Proteins were extracted from CD11b^+^ bone marrow cells, and 50 *μ*g of protein was fractionated by sodium dodecyl sulfate–polyacrylamide gel electrophoresis, transferred to polyvinylidene difluoride membranes, reacted with primary and secondary antibodies, and developed by enhanced chemiluminescence according to standard methods. Antibodies against PDK1, Raptor, and Rictor were purchased from Cell Signaling Technology (Danvers, MA, USA).

### 2.5. CLP Model

The cecal ligation and puncture mice are considered the gold standard for inducing the polymicrobial sepsis. Mice were completely anesthetized with the pentobarbital solution, and a midline abdominal incision was performed. The cecum was ligated at the 1/2 of the distal end and was perforated by sterile needles to induce polymicrobial peritonitis. The number of punctures and the needle size can determine the mortality of CLP mice. The abdominal wall was sutured in two layers and injected subcutaneously with sodium chloride solution for fluid resuscitation. Sham-operated mice underwent a similar protocol without any ligated or punctured after performing laparotomy. We did not observe any mortality in sham animals in all the control and gene knockout mice. The early sepsis was within 5 days after CLP with inflammatory cytokine shock in serum, and the late sepsis was 5-8 days after CLP. To detect the inflammation in the late sepsis, we injected LPS (10 mg/kg) to the mice 7 days after the CLP surgery. The survival and cytokines of the late sepsis were detected after LPS injection.

### 2.6. ELISA

Peripheral blood serum was collected in all the mice at the indicated time, centrifuged at 3,000 × g for 5 min and analyzed following the manufacturer's protocol. The concentrations of TNF*α*, IL-6, IL-1*β*, and IFN-*γ* in the serum were determined by ELISA kits from eBioscience.

### 2.7. Statistical Analysis

Data were analyzed with GraphPad Prism software (GraphPad, San Diego, CA, USA), and all data were showed as mean ± SD. Comparisons between two groups were calculated by Student's *t*-test and one-way ANOVA and with the unpaired two-tailed Student *t*-tests. All the comparisons firstly evaluated the normality by the Kolmogorov-Smirnov test. For the normally distributed data, the comparisons of variables were performed with the unpaired two-tailed Student *t*-tests. When the data were not normally distributed, the comparisons were performed with the Mann–Whitney*U*test for unpaired and paired data. All the comparisons of survival curves were calculated by the Kaplan-Meier method. Differences with *p* values below 0.05 were considered significant. No randomization or exclusion of data points was used.

## 3. Results

### 3.1. Myeloid-Specific PDK1 Deletion Aggravated CLP-Induced Septic Shock

**Figure 1:**
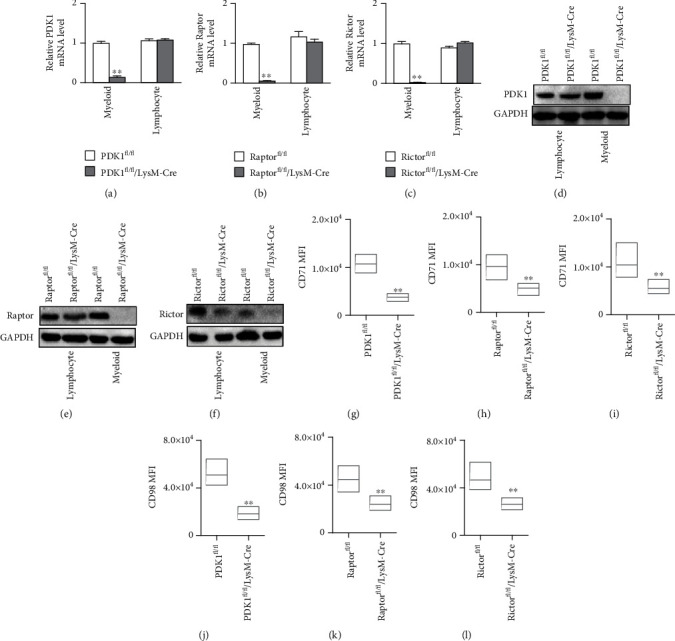
Myeloid-specific PDK1/Raptor/Rictor deletion reduced the metabolism of myeloid cells. (a–c) PDK1, Raptor, and Rictor mRNA in myeloid cells and lymphocyte from PDK1, Raptor, and Rictor myeloid-specific knockout mice were quantified by real-time PCR. Data are presented as the means ± SD. (d–f) PDK1, Raptor, and Rictor protein of the specific knockout mice was detected by western blotting analysis. (g–l) The mean fluorescence index (MFI) of CD71 and CD98 in myeloid cells of indicated mice was analyzed by flow cytometry. Data are representative of three independent experiments.

**Figure 2:**
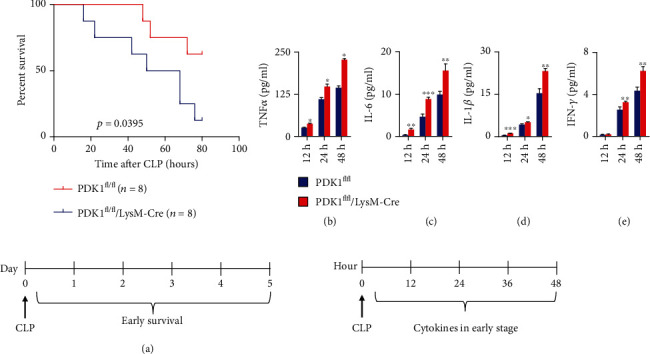
Myeloid-specific PDK1 deletion aggravated CLP-induced septic shock. (a) Littermate PDK1^fl/fl^ and PDK1^fl/fl^/LyzM-Cre mice were subjected to CLP operation, and the survival time was monitored 5 days after the operation. The comparison of survival in different mice was calculated by the Kaplan-Meier method (*p* = 0.0395). (b–e) The measurement of TNF*α*, IL-6, IL-1*β*, and IFN-*γ* secretion by ELISA from serum of PDK1^fl/fl^ and PDK1^fl/fl^/LyzM-Cre mice at 12 h, 24 h, and 48 h after CLP. The comparison of cytokine levels in different mice was calculated by the Mann–Whitney *U* test. ^∗^*p* < 0.05, ^∗∗^*p* < 0.01, and ^∗∗∗^*p* < 0.001. The diagram on the bottom part of the figure was the experimental protocol. PDK1^fl/fl^ and PDK1^fl/fl^/LyzM-Cre mice received the CLP procedure, and mice were monitored for the survival time until 5 days after the operation. The cytokines were collected after the procedure every 12 h for 3 consecutive times.

First, we detected the mRNA (Figures 1(a)–1(c)) and protein expression (Figures 1(d)–1(f)) of PDK1, Raptor, and Rictor in the myeloid cells and nonmyeloid lymphocytes of the mice with related knockout. In these mice, the three proteins were effectively knockout, and the two nutritional receptors CD71 and CD98 representing the cell metabolism were significantly weakened (Figures 1(g)–1(l)). The expression levels of the nutritional markers CD71 and CD98 are commonly used metabolic parameters of immune cells [7, 8]. Then, we analyzed whether the deleted PDK1 on myeloid cells could regulate the early survival of CLP-induced sepsis. The deletion of PDK1 on myeloid cells could significantly increase the early death induced by CLP (*p* = 0.0395) (Figure 2(a)). To further examine the cytokines of the blood in CLP, we performed ELISA assays of the serum of CLP mice. We found that PDK1 deletion could increase TNF*α*, IL-6, IL-1*β*, and IFN-*γ* levels, indicating aggravating inflammation in PDK1 deletion mice (Figures 2(b)–2(e)).

### 3.2. Myeloid-Specific Deletion of mTOR Core Factors Raptor and Rictor Aggravated CLP-Induced Sepsis

**Figure 3:**
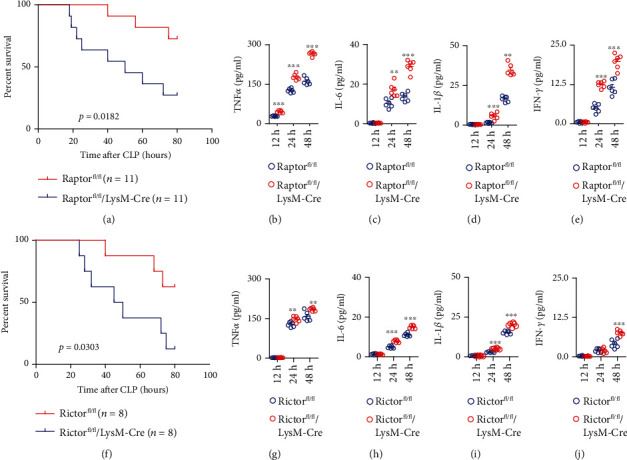
Myeloid-specific deletion of mTOR core factors Raptor and Rictor aggravated CLP-induced sepsis. (a) Littermate Raptor^fl/fl^ and Raptor^fl/fl^/LyzM-Cre mice were subjected to CLP operation, and the survival time was monitored 5 days after the operation. The comparison of survival in different mice was calculated by the Kaplan-Meier method (*p* = 0.0182). (b–e) The measurement of TNF*α*, IL-6, IL-1*β*, and IFN-*γ* secretion by ELISA from serum of Raptor^fl/fl^ and Raptor^fl/fl^/LyzM-Cre mice at 12 h, 24 h, and 48 h after CLP. The comparison of cytokine levels in different mice was calculated by the Mann–Whitney *U* test. ^∗∗^*p* < 0.01; ^∗∗∗^*p* < 0.001. (f) Littermate Rictor^fl/fl^ and Rictor^fl/fl^/LyzM-Cre mice were subjected to CLP operation, and the survival time was monitored 5 days after the operation. The comparison of survival in different mice was calculated by the Kaplan-Meier method (*p* = 0.0303). (g–j) The measurement of TNF*α*, IL-6, IL-1*β*, and IFN-*γ* secretion by ELISA from serum of Rictor^fl/fl^ and Rictor^fl/fl^/LyzM-Cre mice at 12 h, 24 h, and 48 h after CLP. The comparison of cytokine levels in different mice was calculated by the Mann–Whitney *U* test. ^∗∗^*p* < 0.01; ^∗∗∗^*p* < 0.001.

The mTOR was downstream of PDK1 signaling and was a central regulator of the cellular metabolic signaling. So we analyze whether the deletion of mTOR in the myeloid cells could regulate the survival of sepsis. We constructed two of mTOR complex knockout mice in myeloid cells by deleting the core components Raptor (mTORC1) and Rictor (mTORC2). Myeloid deletion of Raptor significantly aggravated CLP-induced early shock (*p* = 0.0182) (Figure 3(a)). Meanwhile, the cytokines such as TNF*α*, IL-6, IL-1*β*, and IFN-*γ* levels rose significantly in mTORC1 deletion mice at 24 h and 48 h, indicating the increased inflammation in these mice (Figures 3(b)–3(e)). Moreover, the myeloid deletion of Rictor significantly aggravated CLP-induced early shock (*p* = 0.0303) (Figure 3(f)). Meanwhile, the levels of TNF*α*, IL-6, and IL-1*β* were significantly enhanced in mTORC2 deletion mice at 24 h and 48 h and IFN-*γ* at 48 h, indicating increased inflammation in these mice (Figures 3(g)–3(j)).

### 3.3. Myeloid-Specific PDK1 Deletion Tuned Down LPS-Induced Inflammation in the Late Stage of Sepsis

Although the improved clinical treatment increased the survival of the patients in the early stage of sepsis [9, 10], they often develop infections leading to death at the later stage of sepsis [11, 12]. This was attributed to immunoparalysis in the later stage by the excessive release of inhibitory molecules, such as the anti-inflammatory cytokines, pathogen recognition signaling suppressors, and the immunomodulatory proteins [13–16].

**Figure 4:**
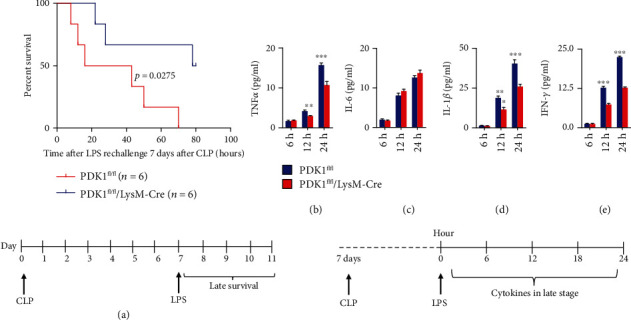
Myeloid-specific PDK1 deletion tuned down LPS-induced inflammation in the later stage of sepsis. (a) Littermate PDK1^fl/fl^ and PDK1^fl/fl^/LyzM-Cre mice were subjected to CLP operation, and 7 days after operation, LPS (10 mg/kg) was peritoneally injected, and the survival time was monitored 5 days after the injection. The comparison of survival in different mice was calculated by the Kaplan-Meier method (*p* = 0.0275). (b–e) The measurement of TNF*α*, IL-6, IL-1*β*, and IFN-*γ* secretion by ELISA from serum of PDK1^fl/fl^ and PDK1^fl/fl^/LyzM-Cre mice at 6 h, 12 h, and 24 h after LPS injection. The comparison of cytokine levels in different mice was calculated by the Mann–Whitney *U* test. ^∗∗^*p* < 0.01; ^∗∗∗^*p* < 0.001. The diagram on the bottom part of the figure was the CLP later stage experimental protocol. PDK1^fl/fl^ and PDK1^fl/fl^/LyzM-Cre mice received the CLP procedure, and 7 days after CLP, LPS was injected to mice. The survival time of all the mice was monitored until 5 days after the LPS injection. The cytokines were collected after LPS injection in the indicated time.

The regulators of early survival might not be coincident with those in the immunosuppressive phase of the late sepsis. We wondered that the regulators of metabolic signaling might play different roles in the late stage of sepsis. We established the CLP model in the PDK1 myeloid-specific knockout mice and the control mice, and seven days later, when the early survival became stable, we injected LPS in these survival mice. We found a completely different role of PDK1 in the late-stage survival. Myeloid deletion of PDK1 significantly tuned down the LPS-induced shock in the late sepsis (7 days after CLP) (*p* = 0.0275) (Figure 4(a)). And the cytokines in the later stage of sepsis were distinct from the early stage. TNF*α* in mouse serum was 100~200 pg/ml at 24 h and 48 h in the early stage of CLP, but it was only 5~10 pg/ml at 12 h and 24 h in the late stage. However, PDK1 deletion could decrease the level of TNF*α* in the late stage of sepsis as the opposite of the early stage (Figure 4(b)). IL-6 in mouse serum was 10~20 pg/ml at 24 h and 48 h in both stages of sepsis, but its level did not change in PDK1 deletion mice in the late stage (Figure 4(c)). IL-1*β* and IFN-*γ* were 15~20 pg/ml and 4~6 pg/ml at 24 h and 48 h in the early stage of CLP but significantly increased to 20~40 pg/ml and 10-20 pg/ml in the late sepsis (Figures 4(d) and 4(e)). Moreover, PDK1 deletion could decrease the levels of IL-1*β* and IFN-*γ* in the late stage of sepsis, as the opposite of early stage. Overall, PDK1 deletion in myeloid cells could significantly alleviate the CLP-induced shock and cytokines in the late stage of sepsis.

### 3.4. Raptor and Rictor Deletion Tuned Up LPS-Induced Inflammation in the Late Stage of Sepsis

**Figure 5:**
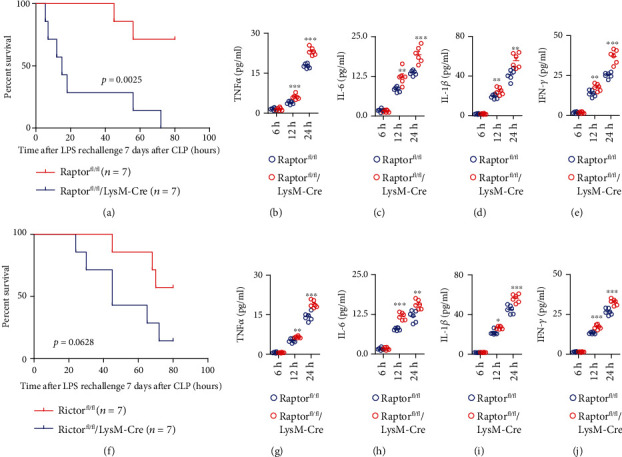
Myeloid-specific Raptor and Rictor deletion tuned down LPS-induced inflammation in the later stage of sepsis. (a) Littermate Raptor^fl/fl^ and Raptor^fl/fl^/LyzM-Cre mice were subjected to CLP operation, and 7 days after operation, LPS (10 mg/kg) was peritoneally injected, and the survival time was monitored 5 days after the injection. The comparison of survival in different mice was calculated by the Kaplan-Meier method (*p* = 0.0025). (b–e) The measurement of TNF*α*, IL-6, IL-1*β*, and IFN-*γ* secretion by ELISA from serum of Raptor^fl/fl^ and Raptor^fl/fl^/LyzM-Cre mice at 6 h, 12 h, and 24 h after LPS injection. The comparison of cytokine levels in different mice was calculated by the Mann–Whitney *U* test. ^∗∗^*p* < 0.01; ^∗∗∗^*p* < 0.001. (f) Littermate Rictor^fl/fl^ and Rictor^fl/fl^/LyzM-Cre mice were subjected to CLP operation, and 7 days after operation, LPS (10 mg/kg) was peritoneally injected, and the survival time was monitored 5 days after the injection. The comparison of survival in different mice was calculated by the Kaplan-Meier method (*p* = 0.0628). (b–e) The measurement of TNF*α*, IL-6, IL-1*β*, and IFN-*γ* secretion by ELISA from serum of Rictor^fl/fl^ and Rictor^fl/fl^/LyzM-Cre mice at 6 h, 12 h, and 24 h after LPS injection. The comparison of cytokine levels in different mice was calculated by the Mann–Whitney *U* test. ^∗^*p* < 0.05, ^∗∗^*p* < 0.01, and ^∗∗∗^*p* < 0.001.

Next, we operated CLP in the Raptor and Rictor myeloid-specific knockout mice and the control mice, and seven days later, when the early survival became stable, LPS was injected in the survival mice. We found a similar function of Raptor and Rictor in the late-stage survival. The myeloid deletion of Raptor, which was mTORC1 deletion, significantly aggravated LPS-induced shock in the later stage of sepsis (*p* = 0.0025) (Figure 5(a)). And the levels of TNF*α*, IL-6, IL-1*β*, and IFN-*γ* in mTORC1 deletion mice were significantly increased in the late stage of sepsis (Figures 5(b)–5(e)). The similar result was found in the mice of mTORC2 deletion. The mTORC2 deletion also aggravated LPS-induced shock in the late sepsis (*p* = 0.0628) (Figure 5(f)). Although the later stage survival in mTORC2 deletion mice was not significant, but the death of knockout mice was delayed. Meanwhile, the cytokine levels increased significantly in the late stage of sepsis (Figures 5(g)–5(j)). Overall, the mTOR complex deletion could aggravate the LPS-induced shock at the late stage of sepsis in the CLP mice, which is completely different with the PDK1 knockout.

To provide a new strategy for septic shock treatment, we used the chemical inhibitors to treat myeloid cells from wild-type mice (Supplementary Figure). As expected, both PDK1 and mTOR inhibitors significantly aggravated the cytokines of myeloid cells in the early stage (Supplementary Figures A–D). In the late stage, the mTOR inhibitor enhanced the cytokines of myeloid cells, but the PDK1 inhibitor relieved the level of TNF*α* and IFN-*γ* (Supplementary Figures E–H).

## 4. Discussion

PDK1/mTOR signaling has been confirmed to regulate the metabolism of tumor [17, 18], T cell [19, 20], B cell [21], and NK cells [22] but has rarely been associated with the myeloid cell metabolism especially in CLP-induced sepsis. In the present study, we found that PDK1 knockout in myeloid cells of CLP mice could aggravate the early septic shock but relieved the secondary inflammation at the late stage of septic shock in CLP-induced sepsis. The different role of PDK1 in the early and late stages of sepsis might be due to the tolerance of the inflammation in the late stage. Immune tolerance is the unresponsiveness of the immune system to the substances or tissues that have the capacity to elicit the immune response in a given organism [23]. The tolerance is induced by prior exposure to that specific antigen without the conventional immune-mediated elimination of them. Immune tolerance is important for normal physiology, such as discriminating self from nonself and preventing overactivation of the immune system upon various microbes. In sepsis, the tolerance can alleviate the tissue damage induced by the overreaction of inflammation. However, which signaling plays the role in the tolerance of myeloid cells was obscure in different infections. Previous reports showed that PDK1 ortholog was necessary in the stress tolerance and in the survival of murine phagocytes [24], indicating that the PDK1 signaling was connected with the tolerance of phagocytes. Moreover, the PI3K/PDK1/Akt pathway could limit the expression of TNF*α* in LPS-stimulated monocytes and ensure transient expression of these inflammatory mediators [25]. The inhibition of Akt could block the trained immunity by monocyte, indicating that the Akt signaling inhibition might block the second inflammation [26]. The PI3K/PDK1/Akt pathway has been indicated to regulate both positively and negatively the expression of NFkB-dependent gene [27–29], which might be the reason of the two type inflammation states in the different stages of sepsis. The PI3K/PDK1/Akt pathway may also regulate different metabolism signaling of myeloid cells in the early and late stages of sepsis. Further work should reveal the mechanism of PDK1 in the myeloid cells of different states.

Although the PDK1 and mTOR pathways perform similar functions in development, proliferation, and survival, the mTORC1/2 knockout in myeloid cells aggravated both the early and the late stages of septic shock. The mTOR signaling was shown to enhance the secondary inflammation of CLP and to regulate the trained immunity of myeloid cells in sepsis. IL-1*β* and IFN-*γ* in mTORC1/2 knockout mice were significantly higher in the second shock than in the first one. Trained immunity was referred as the memory of innate immunity cells such as monocytes/macrophages and the NK cells. During the training, the innate immune cells show an enhanced response to factors produced by pathogenic microbes. Many infections, including bacterial or fungal cells and their components (LPS, *β*-glucan, and chitin) as well as viruses or even parasites, are considered the potent inducers of innate immune memory [30, 31]. Furthermore, the polymicrobial sepsis caused by CLP could also induce the innate immune memory by the second LPS stimuli in the present study. Trained immunity was characterized by a complex interaction among the immunological, metabolic, and epigenetic pathways. The mTOR signaling is the sensor of the metabolic environment and the major regulator of glucose metabolism in activated lymphocytes, so the mTOR signaling could regulate the trained immunity of myeloid cells through metabolism. The mTOR pathways have been indicated to promote the trained monocytes as an epigenetic signal at the promoters of the genes in the mTOR pathway, and the target genes of mTOR were significantly higher in *β*-glucan-trained monocytes [26]. The difference might attribute to the different forms of infections induced by CLP. Moreover, the data from myeloid mTOR deletion mice may reveal the response to the complex interaction of various immune cells *in vivo*. IL-1*β* and IFN-*γ* played crucial roles in the trained immunity, as they function in the initiation and modulation of innate immune responses. Previous work in the vaccine of tuberculosis showed that the enhanced IL-1*β* capacity after BCG vaccination strongly correlated with a lower amount of viremia after yellow fever vaccination. So mTOR signaling might regulate the trained immunity through IL-1*β*.

The trained immunity or the tolerance of innate immunity might attribute to the exposure pattern to the stimulus, the dose, and the exposure time of the stimulus and the subsequent immune responses. For example, a low dose of LPS primes an enhanced immune response to the secondary stimulus, but the high-dose LPS-derived tolerance state allows avoiding an excessive immune response during the secondary stimulation [32, 33]. In the present study, the metabolism signaling of PDK1 and mTOR probably determines the innate immunity in sepsis. PDK1 deletion in myeloid cells could induce the immune tolerance and avoid excessive cytokines during the secondary stimulation. However, the mTORC1/2 knockout mice showed an enhanced immune response to the secondary stimulation. Although PDK1 and mTORC1/2 can both regulate the cellular metabolism, their signal pathways in the innate immunity during the secondary stimulation of sepsis are different. This suggests that the innate immunity is more complex and precise than it has been thought.

The innate immunity is complex enough to induce to the immune memory or tolerance after the primary stimulation, which was once thought to be exclusive to the adaptive immune system. After the first priming, the secondary stimulation could drive the innate immunity toward a heightened or a weakened response. The metabolism signaling of myeloid cells is important for regulating the septic shock in the different stages and might result in the reprogramming of gene transcription associated with the cytokines in sepsis. Further work of immunoregulatory mechanisms about the macrophages or granulocytes might allow the development of new therapeutics. And also, the studies will probably allow a better understanding of the interactions between nonspecific and specific immunity. Better characterization of trained immunity or tolerance would promote the metabolism signaling into the research of immunology, which might offer more effective treatment. Our work will provide a theoretical prospect for the therapy of the septic shock in the different stages.

## 5. Conclusions

Although PDK1 and mTORC1/2 can both regulate the cellular metabolism, the signaling in the innate immunity in the secondary stimulation of sepsis is different. PDK1 deletion in myeloid cells can result in immune tolerance and can help to prevent excessive cytokines during the secondary stimulation, while the mTORC1/2 knockout mice show an enhanced immune response in the secondary stimulation.

## Data Availability

We are committed to providing all the raw data involved in the present study, which can be obtained by contacting Yaxian Kong, email: kongyaxian@ccmu.edu.cn.

## Supplementary Materials

Supplementary materials Supplementary Figure: ex vivo cytokines of myeloid cells were pretreated by chemical inhibitors of PDK1 and mTOR and then were induced by LPS. (A–D) Inhibition of PDK1 and mTOR activity could notably enhance the cytokines of myeloid cells in the early stage of sepsis. Myeloid cells from wild-type mice were pretreated with inhibitors of PDK1 and mTOR for 3 hours and then were incubated with LPS for 3 hours. The medium of the cells with different inhibitors was collected for ELISA. (E–H) Inhibition of PDK1 and mTOR activity in the late stage. Myeloid cells from CLP model mice were pretreated with inhibitors of PDK1 and mTOR for 3 hours and then were incubated with LPS for 3 hours. The medium of the cells with different inhibitors was collected for ELISA. Pharmacological chemical inhibitors GSK2334470 and Torin1 were chosen to suppress PDK1 and mTOR, respectively. GSK2334470 and Torin1 were purchased from Sigma-Aldrich and Tocris Bioscience.
