# Supplementary material for: PDK1/mTOR Signaling in Myeloid Cells Differentially Regulates the Early and Late Stages of Sepsis
Source: Mediators Inflamm. 2020 Jul 25;2020:5437175. doi: 10.1155/2020/5437175 (PMC7397376; doi:10.1155/2020/5437175)
Supplement: Supplementary materials — Supplementary Figure: ex vivo cytokines of myeloid cells were pretreated by chemical inhibitors of PDK1 and mTOR and then were induced by LPS. (A–D) Inhibition of PDK1 and mTOR activity could notably enhance the cytokines of myeloid cells in the early stage of sepsis. Myeloid cells from wild-type mice were pretreated with inhibitors of PDK1 and mTOR for 3 hours and then were incubated with LPS for 3 hours. The medium of the cells with different inhibitors was collected for ELISA. (E–H) Inhibition of PDK1 and mTOR activity in the late stage. Myeloid cells from CLP model mice were pretreated with inhibitors of PDK1 and mTOR for 3 hours and then were incubated with LPS for 3 hours. The medium of the cells with different inhibitors was collected for ELISA. Pharmacological chemical inhibitors GSK2334470 and Torin1 were chosen to suppress PDK1 and mTOR, respectively. GSK2334470 and Torin1 were purchased from Sigma-Aldrich and Tocris Bioscience. [file 5437175.f1.pdf]

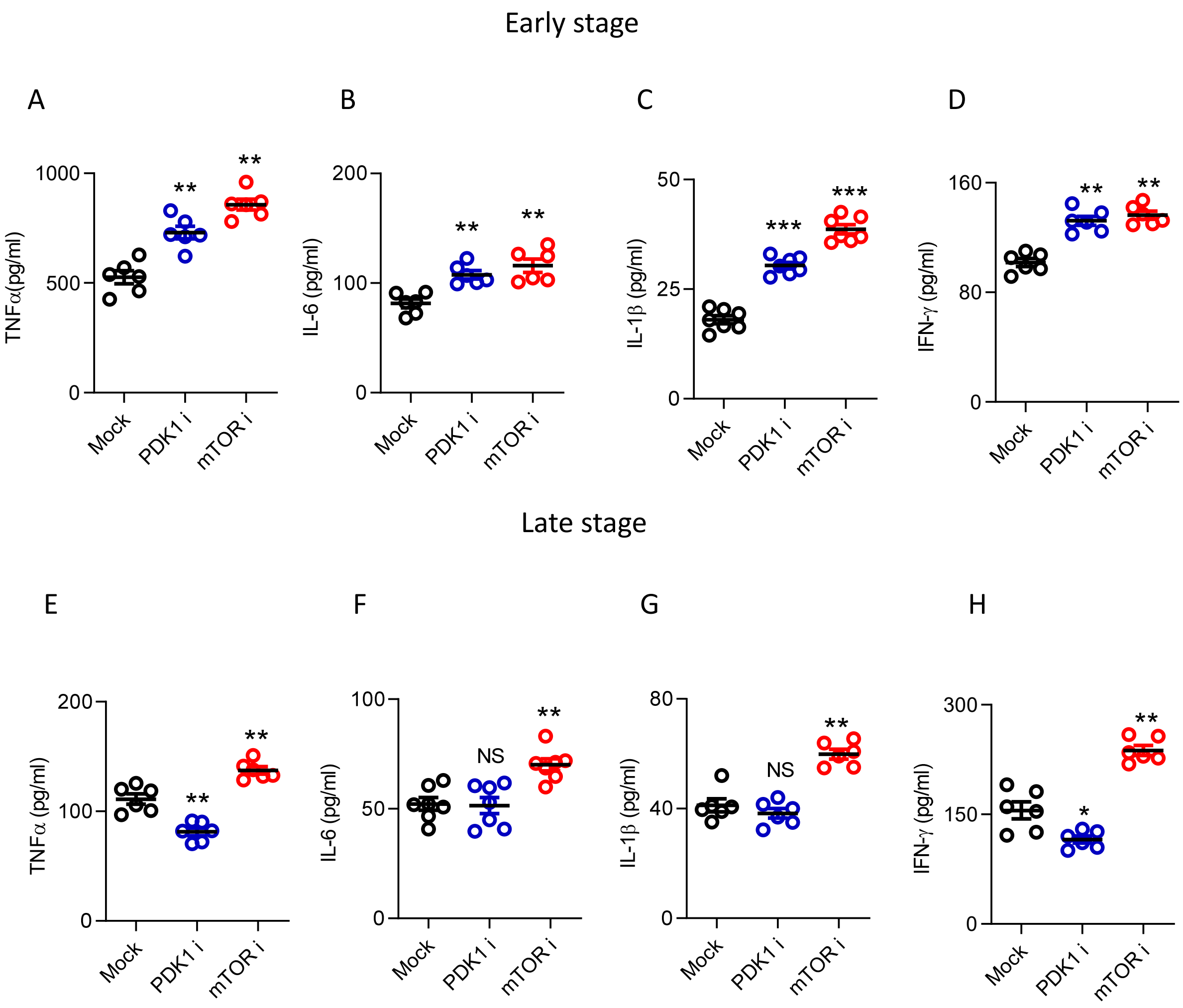

Supplementary Figure. Ex vivo cytokines of myeloid cells were pretreated by chemical inhibitors of PDK1 and mTOR, then were induced by LPS.

(A-D) Inhibition of PDK1 and mTOR activity could notably enhance the cytokines of myeloid cells in the early stage of sepsis. Myeloid cells from wildtype mice were pretreated with inhibitors of PDK1 and mTOR for 3 hours, and then were incubated with LPS for 3 hours. The medium of the cells with different inhibitors were collected for ELISA. (E-H) Inhibition of PDK1 and mTOR activity in the late stage. Myeloid cells from CLP model mice were pretreated with inhibitors of PDK1 and mTOR for 3 hours, and then were incubated with LPS for 3 hours. The medium of the cells with different inhibitors were collected for ELISA. Pharmacological chemical inhibitors GSK2334470 and Torin1 were chosen to suppress PDK1 and mTOR respectively. GSK2334470 and Torin1 were purchased from Sigma-Aldrich and Tocris Bioscience.
